# Culturally adapted psychoeducation for bipolar disorder in a low-resource setting: protocol for a multicentre randomized controlled trial

**DOI:** 10.1192/j.eurpsy.2023.832

**Published:** 2023-07-19

**Authors:** I. Husain, M. Umer, M. Asif, A. Bukhsh, T. Kiran, M. Ansari, H. Aslam, M. Bhatia, F. Dogar, O. Husain, H. A. Khan, A. A. Mufti, B. Mulsant, F. Naeem, H. A. Naqvi, C. De Oliveria, S. Siddiqui, A. Tamizuddin, W. Wang, J. Zaheer, N. Husain, N. Chaudhry, I. Chaudhry

**Affiliations:** 1 Centre for Addiction and Mental Health, Toronto, Canada; 2 Ishrat Husain Pakistan Institute of Living and Learning, Karachi; 3 Liaquat University of Medical and Health Sciences, Hyderabad, Pakistan; 4 Allama Iqbal Medical College/Jinnah Hospital, Lahore; 5 Peoples University of Medical and Health Sciences for Women, Nawabshah; 6 Punjab Institute of Mental Health, Lahore; 7 Balochistan Institute of Psychiatry And Behavioral Sciences, Quetta; 8 Jinnah Medical College, Peshawar; 9 DOW University of Health Sciences, Karachi; 10 National Psychiatric Hospital, Multan; 11 Institute of Psychiatry, WHO Collaborating Centre for Mental Health Research and Training, Rawalpindi, Pakistan; 12 Division of Psychology and Mental Health, School of Health Sciences, Manchester, United Kingdom; 13 Ziauddin University, Karachi, Pakistan

## Abstract

**Introduction:**

Bipolar disorder (BD) is a source of marked disability, morbidity, and premature death. There is a paucity of research on personalized psychosocial interventions for BD, especially in lowresource settings. A previously published pilot randomized controlled trial (RCT) of a Culturally adapted PsychoEducation (CaPE) intervention for BD in Pakistan reported higher patient satisfaction, enhanced medication adherence, knowledge and attitudes towards BD, and improvement in mood symptom scores and health-related quality of life measures compared to treatment-as-usual (TAU).

**Objectives:**

This protocol describes a larger multicentre RCT to confirm the clinical and cost-effectiveness of CaPE in Pakistan.

**Methods:**

A multicentre individual, parallel arm, RCT of CaPE in 300Pakistani adults with BD. Participants over the age of 18, with adiagnosis of bipolar I and II and who are currently euthymic, will berecruited from seven sites including Karachi, Lahore, Multan, Rawalpindi,Peshawar, Hyderabad and Quetta. Time to recurrence will be the primaryoutcome assessed using Longitudinal Interval Follow-up Evaluation(LIFE). Secondary measures will include mood symptomatology, qualityof life and functioning, adherence to psychotropic medications, andknowledge and attitudes towards BD.

**Results:**

Full ethics approval has been received from National Bioethics Committee (NBC) of Pakistan and Centre for Addiction and Mental Health (CAMH), Toronto, Canada. The study has completed sixty-five screening across the seven centres, of which forty-eight participants have been randomised.

**Conclusions:**

A successful trial will lead to rapid implementation of CaPE in clinical practice, not only in Pakistan, but also in other low-resource settings including those in high-income countries, to improve clinical outcomes, social and occupational functioning, and quality of life in South Asian and other minority patients with BD.

**Disclosure of Interest:**

None Declared

